# Obstructive Sleep Apnea, Circadian Clock Disruption, and Metabolic Consequences

**DOI:** 10.3390/metabo13010060

**Published:** 2022-12-30

**Authors:** Mikołaj Malicki, Filip Franciszek Karuga, Bartosz Szmyd, Marcin Sochal, Agata Gabryelska

**Affiliations:** 1 Department of Sleep Medicine and Metabolic Disorders, Medical University of Lodz, 92-215 Lodz, Poland; 2 Department of Pediatrics, Oncology, and Hematology, Medical University of Lodz, 91-738 Lodz, Poland; 3 Department of Neurosurgery, Spine and Peripheral Nerves Surgery, Medical University of Lodz, 90-549 Lodz, Poland

**Keywords:** OSA, circadian disruption, diabetes mellitus, obesity, metabolic complications, microRNA

## Abstract

Obstructive sleep apnea (OSA) is a chronic disorder characterized by recurrent episodes of apnea and hypopnea during sleep. It is associated with various cardiovascular and metabolic complications, including type 2 diabetes mellitus (T2DM) and obesity. Many pathways can be responsible for T2DM development in OSA patients, e.g., those related to HIF-1 and SIRT1 expression. Moreover, epigenetic mechanisms, such as *miRNA181a* or *miRNA199*, are postulated to play a pivotal role in this link. It has been proven that OSA increases the occurrence of circadian clock disruption, which is also a risk factor for metabolic disease development. Circadian clock disruption impairs the metabolism of glucose, lipids, and the secretion of bile acids. Therefore, OSA-induced circadian clock disruption may be a potential, complex, underlying pathway involved in developing and exacerbating metabolic diseases among OSA patients. The current paper summarizes the available information pertaining to the relationship between OSA and circadian clock disruption in the context of potential mechanisms leading to metabolic disorders.

## 1. Introduction

Obstructive sleep apnea (OSA) is a chronic disorder characterized by recurrent episodes of upper airway obstruction during sleep. The oropharynx collapses during apnea or hypopnea episodes, which leads to arousal, sleep fragmentation [1,2], and oxyhemoglobin desaturation. The gold standard in OSA diagnosis is nocturnal polysomnography (PSG), ref. [3] during which the number of apneas or hypopneas per hour of effective sleep is scored, generating the apnea-hypopnea index (AHI), which is used to diagnose and evaluate the severity of OSA.

The most important symptoms are loud snoring, nocturnal awakening, daytime sleepiness, and fatigue [2]. The other possible signs include morning headaches, memory and concentration disruption, night sweats, and gasping [1]. As its symptoms are not specific, OSA remains underdiagnosed. It has been estimated that 82% of men and 93% of women in the United States with OSA have not been diagnosed [4]. Nevertheless, it was noted that the prevalence of OSA is increasing, and there is a clear correlation with the upward trend of the obesity rate. This is in line with obesity being one of OSA’s most important risk factors. According to Punjabi et al., the overall prevalence of OSA ranges from 3% to 7% for adult men and 2% to 5% for adult women in the general population [5]. On the other hand, a cohort study conducted in 2009–2013 on the Swiss population registered that approximately 50% of men and 23% of women experienced at least moderate OSA events [6], showing a continuous increase in OSA prevalence [7].

Furthermore, OSA is strongly associated with multiple comorbidities, such as myocardial infarction, hypertension, arrhythmias, coronary heart disease [8,9,10], stroke [11,12], type 2 diabetes mellitus (T2DM) [13,14,15,16], Alzheimer’s disease [17,18], respiratory disorders [19], nonalcoholic fatty liver disease [20], and immune diseases [21,22,23], as well as pain- and age-related disorders [24,25,26]. The pathological mechanisms responsible for these complications are not yet fully understood. 

Recent data suggest that OSA patients might suffer from circadian rhythm disruption, with the number of studies still growing [27,28]. It is commonly known that mammal organisms, including humans, function according to 24-h patterns called circadian rhythms. Maintaining the mentioned rhythm is possible even without the influence of environmental cues, which proves that it is also driven by internal signals from the organism called core clocks [29]. The daily rhythm can be impaired by extrinsic and intrinsic factors, resulting in circadian misalignment or simply circadian clock disruption [30]. Mentioned terms may include many conditions such as inappropriately timed sleep and wake periods, disordered feeding rhythms, and internal circadian system impairment. The circadian rhythm regulates the activity of almost every organ in the human organism. Therefore, its misalignment leads to various complications, among other metabolic, cardiovascular, and neurodegenerative diseases [29,31,32]. Moreover, multiple conditions, including OSA, can also disrupt circadian cycles. Observations suggest that circadian clock disruption may be significant in the context of the development of OSA complications [27]. Nevertheless, the nature of the relationship between the mentioned disorders is still not fully understood. 

Therefore, we aimed to summarize the available knowledge on the pathways responsible for the development of metabolic disorders in OSA patients in the context of circadian disruption.

## 2. Metabolic Changes in OSA

### 2.1. Diabetes Mellitus and OSA

DM is a group of metabolic disorders characterized by an increased level of blood glucose. The common denominator in all types of this disease is impaired insulin action resulting from its deficiency or elevated tissue resistance. T2DM is the most common type of DM [33]. The pathophysiologic mechanisms leading to T2DM development are not fully understood. Nevertheless, insulin resistance, β—cell dysfunction, imbalanced endocrine activity of the adipose tissue, and gut dysbiosis are among the most frequently reported potential pathologic pathways [34]. The epidemiologic data provide comprehensive data about risk factors for T2DM. They are classically divided into non-modifiable factors such as positive family history, aging, and ethnicity, and modifiable ones, including obesity and low physical activity [35]. T2DM is a vast and still-growing challenge for public health worldwide. In 2017, this disorder affected approximately 6.28% of the world’s population. Furthermore, over 1 million deaths each year result from this disease. Therefore, T2DM is the ninth leading cause of mortality [36].

The correlation between sleep-disordered breathing and impaired glucose metabolism leading to T2DM has been well documented in the literature. The Sleep Heart Health Study revealed that hypoxemia during sleep is associated with glucose intolerance independently of age, body mass index (BMI), gender, and waist circumference [37]. A similar conclusion could be drawn from a newer study based on the Japanese population [38]. A multi-ethnic study of atherosclerosis also confirmed the association between OSA and fasting glucose. The relationship between the mentioned diseases can also be observed on the basis of hemoglobin A1c (HbA1c) level measurements. The HbA1c concentration is a good diagnostic tool for monitoring long-term glycemic control and diabetic complications [39]. Aronsohn et al. performed an investigation that confirmed the above hypothesis. Compared to patients without OSA, the adjusted mean HbA1c was elevated by 1.9% in mild-OSA patients, 1.93% in moderate-OSA patients, and 3.69% in severe-OSA patients [40].

Mahmood et al. estimated, based on 1008 subjects, that the prevalence of T2DM was 30.1% in OSA patients compared to 18.6% in the healthy control group. Furthermore, Kendzerska et al. analyzed the five-year cumulative incidence of T2DM depending on OSA severity. The results showed that in mild OSA patients, this prevalence was 7.5%, with moderate OSA patients showing a prevalence of 9.9%, and severe-OSA patients having a prevalence of 14.9% [41].

The pathomechanism explaining the relationship between OSA and T2DM still needs to be fully understood. This phenomenon is the subject of interest in many articles that propose new molecular pathways in OSA leading to T2DM. Generally, they are associated with impaired glucose metabolism, which is often present in OSA patients [42]. The role of hypoxia-inducible factors (HIFs) such as HIF–1α and reactive oxygen species (ROS) seems to be pivotal [43].

### 2.2. Obesity and OSA

Obesity is an enormous and continuously growing problem for public health. The contemporary situation could be described as a pandemic of adiposity. The prevalence of overweight and obese patients increased by 28% in adults and 47% in the pediatric population worldwide between 1980 and 2013 [44]. The primary definition of obesity is commonly known. It is defined as an excessive accumulation or abnormal distribution of adipose tissue [45]. Obesity can be classified on the basis of BMI [46]. This disorder is associated with many comorbidities, which are among the most significant and studied cardiovascular and metabolic diseases. It is worth noting that in light of the current knowledge, obesity is a heterogeneous syndrome with various phenotypes and complex molecular, immunological, and genetic impairments [47]. However, a detailed description of the mentioned phenomenon is beyond the scope of this review.

The role of adiposity as a cause of various sleep disorders, especially OSA, is well documented in the literature [48,49]. However, a growing number of publications have highlighted the presence of the opposite relationship [50]. Roussard and Cauter proposed that disturbances in sleep and misalignments of circadian rhythms are risk factors for developing lifelong obesity in both adult and pediatric populations [51]. Based on the literature, the most important aspect of sleep disorders in the context of adiposity occurring is an insufficient duration of sleep [52,53]. Patients with such disorders usually have a low-quality diet, which also promotes weight gain [54]. Furthermore, Jung et al. confirmed the role of healthy sleep in energy conservation. Such energy may be used for critical organs in organism, such as the heart, brain, and muscles. Moreover, sleep deprivation triggers decreased energy expenditure and may lead to increased calorie intake. Increased calorie intake promotes the development of obesity [55]. Furthermore, the negative impact of sleep restriction on hormonal balance, including ghrelin, insulin, leptin, and melatonin, has been observed in many studies [51,56,57]. Sleep deprivation is present in OSA due to ineffective sleep caused by arousal during sleep caused by apneas and hypopneas [58]. Thus, mechanisms leading to the development of adiposity observed in sleep deprivation are also present among OSA patients [59].

Obesity can be seen not only as an important risk factor but also as a possible consequence of OSA. There are several probable mechanisms responsible for this relationship. Metabolic changes and OSA-associated circadian rhythm disruptions are possible pathophysiological pathways responsible for this bidirectional association.

## 3. Circadian Clock Organization

The circadian clock is responsible for biological 24-h rhythms in all mammals. Their action results in natural fluctuations of hormones, cytokines, and metabolites in the body’s fluids and tissues [60,61]. Adaptation to the external environment is conditioned by *Zeitgebers* (environmental and social factors that interact with the circadian system and help in the synchronization of biological rhythms [62]) such as mealtimes; physical activity; and, most importantly, high light exposure during the day. This synchronization of endogenous and external signals is called entrainment. Importantly, even in complete darkness, circadian rhythm effects are noted, suggesting that cells have internal rhythm triggers [60,63].

The circadian rhythm is generated by the system of oscillators located in various tissues. The suprachiasmatic nucleus (SCN) in the anterior hypothalamus is the predominant center responsible for self-sustaining circadian oscillations and entrainment phenomena. The main SCN input is the retinohypothalamic tract (RCN), which plays a key role in photic entrainment [63,64,65]. Aside from this, SCN outputs are of great importance because they are responsible for efferent signal transmission. However, the nature of these connections may be surprising. The previous research on hamster models demonstrated that the SCN can influence circadian rhythm via neuroendocrine pathways [66]. The SCN can also communicate with peripheral oscillators located in different tissues and organs, among others in the liver, pancreas, and intestine. They play a vital role in mediating the release of various systemic factors [64].

**Figure 1:**
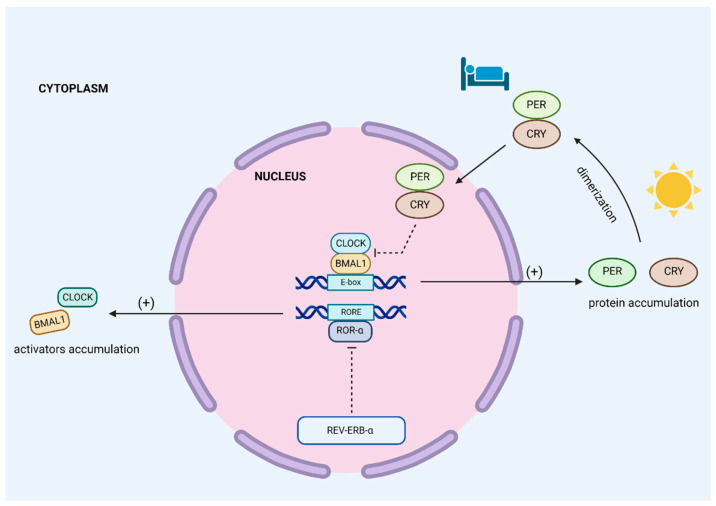
Core circadian clock mechanism. There are two main feedback loops. CLOCK and BMAL1 are transcription factors which act as activators. They work as heterodimers and bind to E-box sequences in DNA, leading to a large number of gene transcriptions, such as *PER*, *CRY*, and *REV-ERB-α.* PER and CRY proteins accumulate during the daily cycle in cells’ cytoplasm. Their highest levels occur before sleep. Following this process, they form a dimerm which is translocated to the nucleus. PER-CRY dimers exhibit repressor activity via the inhibition of CLOCK-BMAL1. This is a basic negative feedback loop that regulates the circadian clock. The second feedback mechanism of the core clock is composed of ROR-α and REV-ERB-α proteins. ROR-α binds to RORE sequences in DNA and promotes the transcription of activators such as CLOCK and BMAL1. REV-ERB-α is a repressor because it inhibits ROR-α activity. BMAL1—brain and muscle ARNT-like 1, CLOCK—clock circadian regulator/circadian locomotor output cycles protein kaput, CRY—cryptochrome, E-box—enhancer box, PER—period protein, REV-ERB-α—nuclear receptor subfamily 1 group D member 1, RORE—ROR response elements, ROR-α—nuclear retinoid-related orphan receptors α.

An in-depth look at the circadian clock at the molecular level can provide an understanding of the interaction mechanisms of circadian rhythms and metabolic disturbances. Each cell’s autonomous, endogenous cycle results from the expression, accumulation, and degradation of the products of “clock genes” [67]. The crucial mechanisms involved in the mammalian circadian clock gene network are positive and negative transcription/translation feedback loops. The first genes that control the circadian rhythm were discovered in *Drosophila* mutants with irregular behavioral cycles. This gene was called *PERIOD*. The analysis of DNA sequences of this region confirmed that RNA transcripts influence biological rhythms. Moreover, the level of individual transcripts was changeable during the day and night cycles [68,69]. The molecular basis of the circadian rhythm is similar, despite the evolutionary distance between insects and mammalians. The genes important for the human clock belong to two groups: repressors, including *PERIOD* (*PER1*, *PER2*, *PER3*) and *CRYPTOCHROME* (*CRY1*, *CRY2*, *CRY3*), which are accumulated during the day cycle, and activators, which are *CLOCK* and *BMAL (BMAL1, BMAL2).* They work as heterodimers; CLOCK/BMAL regulates the daytime expression of PER and CRY proteins by connecting to the promoter regions (E-boxes) of repressor clock genes, resulting in the accumulation of PER and CRY proteins, which form dimers. In this form, they translocate to the cellular nucleus, where these protein complexes stop their transcription. The repression of CLOCK/BMAL occurs during the night; it is the negative limb of the main feedback loop. Furthermore, the degradation of PER/CRY is crucial for starting the new cycle [70,71]. Also necessary for proper circadian clock physiology are regulatory factors such as NPAS2 (a paralogue of CLOCK, which exhibits the same function, creates the BMAL-NPAS2 complex [72]). The creation of the BMAL-CLOCK complex leads to the increased expression of regulators, including RORα or REV-ERB-α. The latter is a DNA-binding protein that suppresses RORα activity [72,73] (see Figure 1).

## 4. Circadian Clock Disruption in the Context of Metabolic Changes

### 4.1. General Impact of Circadian Clock Disruption on Metabolism

Almost 40% of human genes exhibit circadian oscillations in their transcript [74]. This leads to the observation of 24-h cycle-dependent fluctuations in metabolic parameters such as glucose, insulin, and leptin [75]. In line with these results are epidemiological studies showing that human clock disruption leads to significant metabolic consequences. Namely, shift-working (an external factor resulting in clock misalignment) stimulates the development of obesity and increases the risk of insulin resistance, T2DM, and the occurrence of cardiovascular complications [76,77].

A particularly interesting phenomenon in the context of human biological clock disruption is circadian misalignment. This refers to discrepancies between the central circadian pacemaker and the 24-h behavioral rhythm, influenced by factors such as light and dark or feeding and fasting cycles [29]. Morris et al. performed a randomized crossover study and revealed that circadian misalignment causes reduced glucose tolerance, which can lead to diabetes [78].

The link between circadian clock disruption and metabolic disorders was also confirmed in research on animal models. Turek et al. revealed that homozygous CLOCK-mutant mice were susceptible to obesity [79]. Furthermore, such mice also developed hyperphagia in early life and metabolic syndromes, including hyperleptinemia, hyperlipidemia, hyperglycemia, and hypoinsulinemia [79,80]. Additionally, similar consequences developed models with *BMAL1-* or *PER2*-knock-out mutations and *CRY1*/*CRY2* double-knock-out mutations [81,82,83,84].

The above link was corroborated via an analysis of human polymorphisms in clock genes, which exhibited a predisposition towards obesity and metabolic syndrome. Indeed, polymorphisms in CLOCK and BMAL1 were associated with the development of metabolic syndrome. Furthermore, CLOCK gene variants have also been associated with the risk of obesity [85,86]. Woon et al. reported that two BMAL1 haplotypes were responsible for T2DM and hypertension [87]. Additionally, polymorphisms in CRY2 have been related to increased fasting glucose, elevating the risk of T2DM development [88]. However, the positive correlation between polymorphism in REV-ERB-α and obesity has been confirmed only for the male population [89].

### 4.2. Glucose Metabolism

Many factors have an impact on glucose levels. However, insulin and glycogen are the main regulators [90]. The effect of the circadian clock on glucose homeostasis is observed in healthy animals and humans as a diurnal rhythm affecting the glucose level under a regular light/dark cycle, with a higher concentration at waking and during sleep [91,92]. The mentioned fluctuations are independent of eating behaviors [93]. Moreover, it has been reported that both glucose tolerance and insulin levels also exhibit diurnal fluctuations. Additionally, oral glucose tolerance is disrupted in the evening rather than the morning. It has been suggested that decreased insulin secretion and impaired insulin susceptibility in the evening are responsible for these observations [94,95]. Similarly, the expression of factors involved in the glucose metabolic pathway also presents circadian fluctuations, for example, in GLUT2 (a hepatic glucose transporter) and in glucokinase (an enzyme activated in glycolysis, the main pathway of glucose catabolism [96]). In agreement with the mentioned findings, a study conducted on rat models confirmed the role of the SCN in maintaining physiological fluctuations of glucose levels [97]. Moreover, the glucose circadian rhythm was disrupted in animal models with SCN impairment. Hogenboom et al. performed a post-mortem analysis of brain tissues from T2DM patients [98]. They revealed that the number of neurons in the SCNs was significantly decreased [98]. These results are in agreement with previous findings and suggest that the SCN disruption may be significant in T2DM pathogenesis due to impaired clock machinery. Nevertheless, a network of interactions with the SCN still needs to be clarified. However, several possible pathways responsible for the diurnal rhythm of glucose have been proposed.

As glucocorticoids regulate various aspects of glucose metabolism and exhibit a circadian rhythm, they are one of the most significant links between circadian disruption and glucose metabolism impairment [99]. Glucocorticoids are steroid hormones that regulate various aspects of glucose metabolism. They accelerate gluconeogenesis in the liver and reduce glucose absorption by skeletal muscles and adipose tissue cells. They antagonize the insulin response and exhibit a hyperglycemic effect [100]. Buijs et al. revealed an interplay between the central clock pacemaker and glucocorticoids secretion [101]. Namely, the SCN regulates corticotropin-releasing hormone (CRH) secretion in the paraventricular nucleus. The CRH promotes the release of adrenocorticotropic hormone (ACTH) from the anterior lobe of the pituitary gland [101]. Moreover, the systemic source of circulating glucocorticoids is also regulated by the circadian clock located in the adrenal glands [102,103]. Moreover, Bright et al. reported that cortisol blockage is ineffective in suppressing elevated morning glucose levels in diabetic patients [104]. Understanding the relationship between the circadian clock, glucocorticoids, and diurnal glucose fluctuations is still a challenge.

Growth hormone (GH) is another factor that impacts metabolism. This substance, released by the pituitary gland, accelerates lipolysis and decreases glucose utilization [105]. It has been reported that GH exhibits a diurnal rhythm [106,107]. Moreover, research on shift workers revealed that 24-h-pattern disruption results in unpredictable pulses of GH secretion during the light/waking period [108,109,110]. Further investigation uncovered that the SCN and the peripheral clock in somatotrophic pituitary cells regulated the circadian rhythm of GH. The activity of GH is strongly associated with insulin-like growth factor 1 (IGF-1) production. GH promotes the release of IGF-1 in the liver. An elevated concentration of IGF-1 suppresses GH secretion, creating a negative feedback loop [111,112]. Liu et al. investigated mice with liver-specific GH-receptor knockout mutations, which exhibited decreased levels of IGF-1 and increased glycemia and blood insulin concentrations [113]. IGF-1 is also associated with circadian fluctuations. Interestingly, there are differences between animal and human subjects. In rats, IGF-1 levels are higher during sleep and reduced during the waking period [114,115]. The situation is the opposite in humans, and blood IGF-1 peaks in the morning after nightfall [116]. Thus, GH may contribute to diurnal glucose rhythm.

Another pathway responsible for glucose fluctuations may be related to thyroid hormones. Its biological activity is associated with tetraiodothyronine (T4), triiodothyronine (T3), and diiodothyronine (T2) [117]. Secretion of the mentioned hormone is regulated by thyroid-stimulating hormone (TSH) released by the anterior pituitary gland and thyrotropin-releasing hormone (TRH) produced in the hypothalamus [118]. Both T3 and T4 suppress TRH and TSH, creating the hypothalamus–pituitary–thyroid axis [119]. The association between thyroid hormones and glucose homeostasis is well-documented in the literature [120,121]. Hyperthyroidism results in reversible hyperglycemia [121,122] and promotes gluconeogenesis, which may be recognized as a cause of the hyperglycemic effect of this disorder [123]. Similarly, a study of the Korean population revealed a partially positive correlation between free thyroxin and fasting glucose levels [124]. The impact of the circadian clock on thyroid hormones is observed in their diurnal fluctuations [125,126]. Fahrenkrug et al. showed that the excision of the pituitary gland in rats disrupted thyroid hormone fluctuations [127]. However, the expression of clock genes in the thyroid remained intact, suggesting that the diurnal rhythm of T3 and T4 is regulated by the SCN or the pituitary gland, rather than the peripheral clock located in the thyroid [127].

Recent discoveries have led to an interest in melatonergic system activity as a potential network regulating diurnal glucose fluctuations [128]. As mentioned before, melatonin is a pleiotropic hormone secreted by the pineal gland, regulating both circadian and seasonal rhythms in the human body [129]. The secretion of melatonin is regulated by the SCN and by light intensity [130]. Furthermore, it was observed that genetic variants of the *MTNR1B* (the gene which encodes the melatonin MT2 receptor) significantly affect fasting plasma glucose levels and are associated with the risk of T2DM development [131,132,133,134]. Furthermore, removal of the animal pineal gland resulted in altered melatonin oscillations and impaired the circadian rhythm of glucose tolerance and insulin sensitivity [135]. Studies conducted in rodent models revealed that MT2 and MT1-knock-out mice exhibited increased insulin secretion [136,137,138]. However, reduced plasma insulin levels and increased glucose concentrations in MT1-knock-out blood were observed [137]. Recent research showed that MT1-knock-out mice manifest a disordered ability to metabolize glucose [136]. Molecular interactions responsible for the relationship described above are still controversial. Nevertheless, it seems that melatonin can regulate glucose levels via a negative effect on insulin secretion.

Moreover, orexins (hypocretins) exhibit the potential for glucose level regulation. They are a group of neuropeptides released by a limited number of neurons in the lateral and perifornical areas of the hypothalamus [139]. Orexins stabilize sleep and wakefulness [140]. Interestingly, they exhibit diurnal fluctuations in cerebrospinal fluid, with a peak during waking, but rhythmicity in blood has not been observed [141,142,143]. Orexins play an essential role in sleep stability because their decreased level in cerebrospinal fluid was established as one of the diagnostic markers for narcolepsy-cataplexy (a primary sleep disorder characterized by excessive daytime sleepiness, hypnogogic hallucinations, etc. [144]). Additionally, studies on animal models revealed an association between the above-described peptides and glucose metabolism [145,146,147]. Tsuneki et al. reported that physiological glucose rhythm was disrupted in orexin knock-out mice, suggesting the orexin system’s importance in metabolism [145]. Moreover, they showed that intracerebroventricular administration of orexin A during the waking period significantly increased blood glucose levels. However, daily glucose levels were reduced in both healthy and diabetic mice. This neuropeptide also regulates the expression of gluconeogenic genes, which suggests that orexin A can promote diurnal glucose rhythm via the regulation of gluconeogenesis [145]. Additionally, it has been reported that orexin stimulates insulin secretion in normal and diabetic conditions [146,147,148]. Zhang et al. revealed that orexin A promotes glucose uptake via increasing GLUT4 expression [149]. Thus, the abovementioned neuropeptides can reduce blood glucose levels and manifest a beneficial impact on DM development. It was also reported that orexin A administration in rats with T2DM resulted in improved glucose control, elevated insulin sensitivity, and reduced β-cell loss [150]. Moreover, the expression of the orexin-A receptor was observed to be decreased in mice with T1DM [151].

It is worth mentioning that hypocretin/orexin-mediated pathways may be associated with ghrelin. This is a multifunctional peptide secreted by the X/A-like cells in the stomach [152]. It is known that ghrelin stimulates GH secretion (thus, ghrelin-mediated pathways may be associated with those including GH and IGF-1 described above) [153]. This hormone is also related to the circadian clock. Szentirmai et al. revealed that the microinjection of ghrelin into the hypothalamic area induced wakefulness in rats [154]. It has been reported that ghrelin exhibits a diurnal rhythm with a peak during the evening. Moreover, circadian misalignment results in increased postprandial activity [155]. Ghrelin is strongly associated with metabolism. There are two forms of this hormone in the human organism, acylated and deacylated [156]. It has been suggested that they exhibit the opposite effect on metabolism [157], which may be a factor that hinders research. It has been reported that unacylated ghrelin regulates feeding via the activation of orexin-A-immunopositive neurons [158]. This hormone may also affect glucose homeostasis via the regulation of insulin secretion. The majority of studies report that ghrelin suppresses insulin secretion. However, some researchers have observed stimulation or simply no effect on insulin release [159]. Sun et al. revealed that the ablation of ghrelin in diabetic mice led to an increased release of insulin, reduced blood glucose levels, and the enhancement of glucose tolerance [160]. Therefore, this study suggested that ghrelin had a suppressive effect on insulin production. It has been suggested that various observations of the effect of ghrelin on insulin may be associated with glycemic conditions [159]. Thus, ghrelin may be potentially involved in regulating the diurnal glucose rhythm. Nevertheless, further research is required to confirm this concept.

Circadian fluctuations in glucose levels are also apparent in relation to clinical consequences. A good example is daily doses of insulin administered to diabetic patients [161]. Additionally, it may be related to the “dawn phenomenon” (DP) in patients suffering from DM. This condition occurs in 20% to 90% of T1DM and 6% to 90% of T2DM patients [162]. It can be defined as spontaneous early-morning hyperglycemia without hypoglycemia during the night. The post-breakfast hyperglycemia is described as “extended DP”. It is a more robust feature of DP [163]. Interestingly, Huang et al. showed that poor sleep quality in diabetic patients is associated with an increased DP and lower expression of *BMAL1* and *PER1* genes [164]. The molecular mechanisms responsible for the relationship between circadian clock disruption and DP are still not fully understood. However, it has been suggested that REV-ERB is involved in the pathogenesis of extended DP because this group of patients exhibits the differential temporal expression pattern of this transcriptional factor [165]. 

### 4.3. Lipid Metabolism

Fatty acid synthesis and their beta-oxidation play an important role in lipid metabolism. Moreover, several stages of the mentioned process are related to circadian clocks. For example, ATP citrate lyase (ACLY) is an essential enzyme for citrate/palmitate shuttle effectiveness, the mechanism responsible for the transport of mitochondrial acetyl-CoA to the cytoplasm. It has been reported that the circadian peak of ACLY is associated with the feeding cycle [96]. Another site where the circadian cycle is combined with lipid metabolism is fatty acid β-oxidation. This correlation is conditioned by the daily rhythms of carnitine palmitoyl transferase 1 (CPT1) and CPT2 concentrations. These enzymes are required to introduce acyl groups into the mitochondria. The β-oxidation process depends on their level in this cellular structure [96]. Additionally, REV–ERB-α plays a role in lipid balance. This transcriptional repressor is strongly associated with histone deacetylase 3 (HDAC3) in the context of lipid metabolism regulation. A low concentration of REV–ERB-α during the active or feeding cycle triggers a decreased relationship of HDAC3 with the liver genome. The result is an increase in lipogenesis. A high level of REV–ERB-α, which is characteristic of inactive or fasting time, leads to a reversed process. Thus, it reduces lipogenesis. In a study conducted by Feng et al., mice with genetically deleted REV–ERB-α exhibited liver steatosis [166].

Numerous studies have reported the impact of the circadian clock on phospholipid homeostasis. It was discovered in a chick model that enzymes involved in phospholipid metabolism, such as lysophospholipid acyltransferases, phosphatidate phosphohydrolase, and diacylglycerol lipase, exhibit diurnal rhythms [167]. Retinal phospholipid-synthetic enzymes in rats showed similar variations [168]. Other investigations based on animal models in which metabolomic measurements in the blood were performed confirmed that peaks in phospholipid levels were associated with circadian rhythms [169].

Several studies have revealed that similar variations in phospholipid contents occur in human organisms. Ruf et al. examined samples of human mucosa cells from twenty healthy subjects to determine the seasonal and daily rhythmicity of various phospholipid fatty acid proportions. The authors reported that significant circadian rhythms were observed in 11 of the 13 fatty acids [170]. A much larger set of epidemiological data confirmed this relationship. A study based on 162 subjects showed differences in circulating lipid components (phospholipids, total cholesterol, and high-density lipoproteins) dependent on the diurnal cycle [171].

### 4.4. Bile Acids

Bile acids are steroid acids that play a vital role in energy metabolism by regulating glucose, lipids, and energy expenditure [172]. They are synthesized in the liver from cholesterol. Newly produced bile acids are secreted into the bile; next, they are transported to the small intestine, where they emulsify lipids, cholesterol, and fat-soluble vitamins. These nutrients are delivered to the liver in the form of lipoproteins, where they are further metabolized [173]. Disruptions in the homeostatic balance of bile acids are associated with cholestatic liver diseases, obesity, and diabetes mellitus [174].

**Figure 2:**
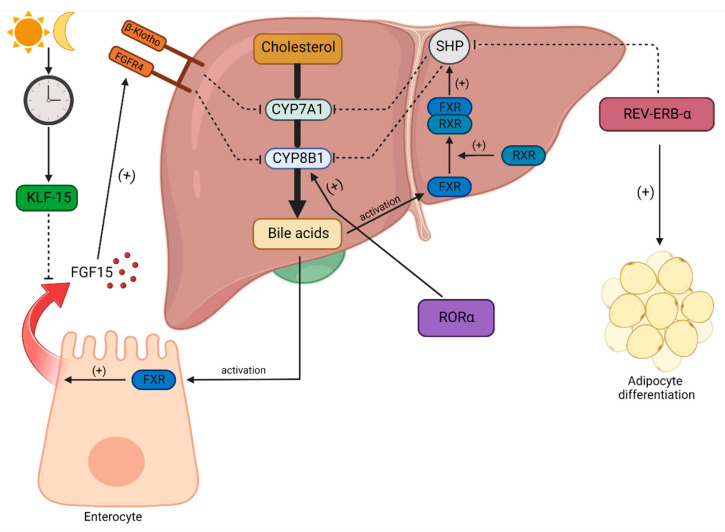
Impact of the circadian clock on lipid and bile acid homeostasis. Cholesterol is converted into bile acids in the biochemical pathway in hepatocytes. The main enzymes of this pathway are cholesterol 7 alpha-hydroxylase (CYP7A1) and sterol 12-alpha-hydroxylase (CYP8B1). CYP8B1 is stimulated by one of the circadian clock proteins, orphan receptor α (RORα). The production of bile acids is regulated by a negative feedback loop. Bile acids activate the nuclear farnesoid X receptor (FXR). It binds to the retinoid X receptor (RXR) and forms a heterodimer. The FXR-RXR complex activates a small heterodimer partner (SHP). The SHP protein inhibits CYP7A1 and CYP8B1 enzymes and decreases the production of bile acids. The circadian clock can influence the bile acid synthesis pathway via SHP. Specifically, REV-ERB-α inhibits SHP. Thus, REV-ERB-α promotes bile acid synthesis. Moreover, it stimulates adipocyte differentiation. Some bile acids are transported into the small intestine. They activate the FXR in enterocytes, resulting in the release of fibroblast growth factor 15 (FGF15). FGF15 promotes the fibroblast growth factor receptor-4/β-Klotho (FGFR4/β-Klotho) complex, inhibiting CYP7A1 and CYP8B1. This is the second negative feedback loop regulating bile acid synthesis. Day and night cycles can influence bile acid synthesis via the circadian regulation of Krupple-like factor 15 (KLF15) production. KLF15 restrains the release of FGF15 in enterocytes. CYP7A1—cholesterol 7 alpha-hydroxylase, CYP8B1—sterol 12-alpha-hydroxylase, FGF15—fibroblast growth factor 15, FGFR4—fibroblast growth factor receptor-4, FXR—nuclear farnesoid X receptor, KLF15—Krupple-like factor 15, REV-ERB-α—nuclear receptor subfamily 1 group D member 1, ROR-α—nuclear retinoid-related orphan receptors α, RXR—retinoid X receptor, SHP—small heterodimer partner, β-Klotho—β-Klotho.

The concentration of circulating bile acids is associated with day and night rhythms [175]. Circadian rhythms play a pivotal role in the regulation of bile acid homeostasis. The main enzyme responsible for the conversion of cholesterol into bile acids is 7α-hydroxylase (CYP7A1). This microsomal enzyme exhibits a diurnal rhythm of mRNA expression. In the human organism, the level of CYP7A1 is increased during the daytime with peaks at 1:00 pm and 9:00 pm. The intensification of its expression declines at night [176]. It has been suggested that the diurnal rhythm of CYP7A1 and CYP8B1 (sterol 12αhydroxylase, another enzyme crucial for bile acid synthesis) is the result of molecular clock mechanisms [177]. Pathak et al. give an example of direct interactions between clock genes and enzymes related to bile acid levels. They reported that retinoic acid-related orphan receptor α (RORα) strongly induces CYP8B1. Due to this process, RORα increases the concentration of 12α-hydroxylated bile acids in serum [178]. Duez et al. observed that REV—ERB-α-deficient mice presented a lower intensification of synthesis and impaired bile acid excretion. The subsequent analysis confirmed that REV–ERB-α targets small heterodimer partner (*SHP*) and *E4BP4* genes. The final effect of this loop is the stimulation of CYP7A1 transcription [179]. It has also been reported that CLOCK can direct the stimulation of SHP (a nuclear receptor that binds to and inhibits the CYP7A1 promoter factor) [180]. Eggink et al. described the synthesis of other complex molecular pathway-regulating bile acids by a negative feedback loop associated with circadian rhythms. Specifically, bile acids can activate the nuclear farnesoid X receptor (FXR). Then, activated FXR binds to the retinoid X receptor (RXR) and forms a heterodimer. The FXR-RXR complex promotes SHP expression, leading to CYP7A1 and CYP8B1 inhibition in hepatocytes. There are much more factors that play an important role in this loop. The essential one is certainly fibroblast growth factor 15 (FGF15). It acts as an FXR-regulated hormone that activates the FGFR4/β-Klotho complex responsible for inhibiting the abovementioned CYP genes [177,181]. Han et al. showed that FGF15 is negatively regulated by Krupple-like factor 15 (KLF15), which exhibits a circadian rhythm. In sum, bile acids can regulate their expression by means of a negative feedback loop mediated by FXR; this mechanism is not free from the influence of diurnal rhythms due to its association with the KLF15—FGF15 signaling axis [182] (see Figure 2). Ma et al. examined mice with the genetic ablation of *PER1* and *PER2* genes and observed an increased level of aspartate aminotransferase (AST), a biomarker of liver damage and elevated serum bile acids [183]. The authors concluded that circadian clock disruptions lead to the dysregulation of bile acids and cholestatic disease development [183]. Yu et al., in a review, summarized research on the topic, showing that impaired bile acid homeostasis triggered by circadian rhythm disruption leads to cholestatic and metabolic diseases. It has even been proposed that developing a therapeutic target of the circadian rhythm might be effective in such a case. However, the available data are insufficient for this purpose; therefore, more studies are needed on this topic [184].

### 4.5. NAD+

Nicotinamide adenine dinucleotide (NAD+) acts as an electron carrier in organisms, so it is involved in various processes, such as metabolic pathways, transcription, signaling, and cell survival. Therefore, NAD+ homeostasis is an important parameter with a clear impact on metabolism regulation [185]. It has also been reported that the NAD+ concentration is correlated with metabolic efficiency and cellular function. An increased level of NAD+ in mammalian tissue is a common response to exercise and decreased calorie intake [186]. The level of NAD+ is determined by the balance between consumption and biosynthesis [185]. Consumption is the conversion of NAD+ to nicotinamide by poly ADP-ribose polymerases (PARPs), sirtuins, and the cluster of differentiation 38 (CD38). The biosynthesis pathway is complex, but the main enzyme involved in this process is nicotinamide phosphoribosyltransferase (NAMPT). It converts Nam into nicotinamide mononucleotide (NMN). Subsequently, NMN serves as a substrate for NAD+ production, which occurs in several steps [185].

The circadian clock is associated with NAD+ homeostasis. This electron carrier presents diurnal oscillations, driven by the core clock [187]. Circadian oscillations of NAD+ may lead to similar variations in mitochondrial fatty acid oxidation and oxygen consumption in hepatocytes and skeletal muscle cells [188]. It has been observed that the rhythmic expression of NAMPT occurs in *PER2* phase expression in many mouse tissues [189]. Ramsey et al. revealed that the inhibition of NAMPT promotes the oscillation of PER2 by releasing CLOCK-BMAL1 from SIRT1-dependent suppression [187].

There is a functional link between NAD+ concentrations and the metabolic activity of cells via the action of SIRTs. These are NAD+-dependent enzymes responsible for removing acyl groups from lysine in histone and other proteins. Moreover, we have distinguished several isoforms of these enzymes in mammalian cells [190]. The molecular association of SIRT1 and CLOCK was assessed. The link was confirmed via genetic ablation of the *SIRT1* gene and the pharmacological inhibition of this deacetylase. In both cases, a disruption in the circadian clock was the final result. SIRT1 impacts many signaling pathways and genome stability via its targets, including histones (H3 and H4), as well as non-histone proteins such as p53, FOXO3, PGC—1α, and LXR [191].

## 5. OSA and Circadian Clock Disruption

Several studies have discussed the link between OSA and circadian clock disruption [192]. Additionally, the risk of both cardiovascular and central nervous system disorders in OSA patients is quite similar to those of patients with disrupted sleep, only in the absence of hypoxic episodes [193]. This further suggests that both OSA and circadian clock disorders may be associated with similar molecular pathways. Interestingly, the analysis of disordered rhythms of transcriptomes and metabolites in samples from OSA patients has been proposed as a novel diagnostic biomarker for this disorder [27]. Although here we have focused on the molecular pathways responsible for clinical manifestations of OSA and its complications, the role of other factors should be mentioned. One of them is arousal from sleep, which can be spontaneous or induced by movements or abnormal breathing. In OSA patients, they can be triggered by ventilatory efforts in response to hypoxia or hypercapnia [194]. Interestingly, arousal may exhibit bidirectional effects on circadian clock disruptions, OSA severity, and metabolic complications via increased sympathetic activity. Clinically, it has been reported that the “arousal index” is an even better predictor of sympathetic activation during wakefulness and of cardiovascular complications than AHI or nocturnal hypoxemia levels [195,196].

**Figure 3:**
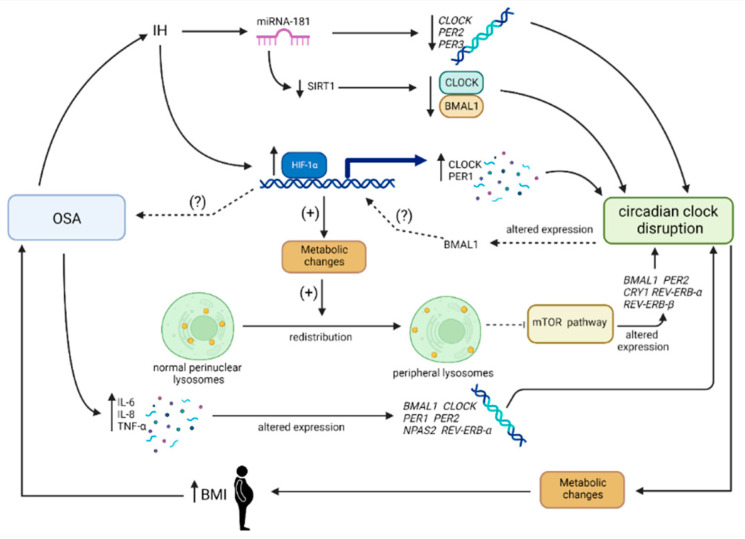
The bidirectional link between OSA and circadian clock disruption. Obstructive sleep apnea (OSA) is associated with the occurrence of recurring intermittent hypoxia (IH) episodes. In such conditions, a *miRNA-181* family, which is oxygen-sensitive, is overexpressed. This leads to the silencing of miRNA-181-targeted genes, including, among others, *CLOCK*, *PER2*, and *PER3.* Impaired clock gene fluctuations result in circadian clock disruption. Furthermore, miRNA-181 also targets the *SIRT1* gene. SIRT1 deficiency leads to decreased CLOCK-BMAL1 levels, which triggers impaired circadian gene transcriptions and, finally, the development of circadian clock disruption. In oxygen-limited conditions, hypoxia-inducible factor-1α (HIF-1α) is upregulated. This results in increased levels of CLOCK and PER1 proteins. As mentioned above, changes in circadian clock gene products fluctuations leads to circadian clock disruptions. The overexpression of HIF-1α also generates metabolic changes. Subsequently, the altered spatial lysosome distribution in cells inhibits the mechanistic target of the rapamycin kinase (mTOR) pathway. It also induces changes in circadian clock gene levels. The HIF-1α-related pathway is most likely bidirectional. Circadian misalignment results in altered BMAL1 expression. This triggers HIF-1α upregulation, which probably promotes OSA development. Another pathway is associated with low-grade inflammation. OSA stimulates the overproduction of interleukin (IL) 6 and 8, as well as tumor necrosis factor-α (TNF-α). The basic opposite pathway is associated with metabolic changes resulting from a disordered circadian clock. This promotes an increase in body mass index (BMI), an established risk factor for OSA development. BMAL1—brain and muscle ARNT-like 1, BMI—body mass index, CLOCK—clock circadian regulator/circadian locomotor output cycles protein kaput, CRY1—cryptochrome 1, HIF-1α—hypoxia-inducible factor-1α, IH—intermittent hypoxia, IL-6—interleukin 6, IL-8—interleukin 8, NPAS2—neuronal PAS domain protein 2, miRNA-181—micro RNA-181, OSA—obstructive sleep apnea, PER1—period protein 1, PER2—period protein 2, PER3—period protein 3, REV-ERB-α—nuclear receptor subfamily 1 group D member 1, SIRT1—sirtuin 1, TNF-α—tumor necrosis factor-α.

The relationship between OSA and circadian clock disruption is most likely bidirectional. However, the underlying causes of this phenomenon remain unclear. Nevertheless, a growing number of studies have highlighted the crosstalk between the circadian clock and hypoxia, the key symptom of OSA [27,197,198] (see Figure 3). Hypoxia signaling is crucial in maintaining homeostasis in conditions with limited oxygen [199]. Intermittent hypoxia (IH), which is characteristic of OSA [20], activates hypoxia-inducible factors, which are pivotal factors in oxygen metabolism [20]. They are heterodimeric complexes composed of two subunits: α (HIFα) and β (HIFβ) [200]. The subunit α is oxygen-sensitive [201]. In hypoxic conditions, HIFα is translocated into the nucleus, creating an active heterodimeric complex with HIFβ. The HIFα:HIFβ complex binds to hypoxia-responsive elements (HREs) on its target genes. The final step is their transcriptional upregulation [202]. The most studied molecule among the mentioned factors is HIF-1 [203]. Adamovich et al. revealed that oxygen levels exhibit daily fluctuations in the blood and tissue of rodents. Furthermore, the abovementioned oxygen rhythm has the ability to reset clocks in an HIF1α-dependent manner [204]. They additionally observed that HIFα has an impact on clock gene expression [204]. A probable explanation for the mentioned relationship is that clock genes, including *CRY1*, *CRY2*, *PER1*, and *CLOCK*, have both E-box and HRE motifs in their promotors [205,206]. As mentioned above, HRE is targeted by the HIF transcriptional factor, so it has the ability to upregulate such genes. This hypothesis was corroborated by Chilov et al., who uncovered an increased level of PER1 and CLOCK proteins in mouse brain cells under hypoxic conditions [207]. In agreement with these findings, OSA patients exhibited HIF-1α upregulation [208]. Further research revealed that this transcriptional factor enhances PER2 expression [209]. In light of this knowledge, the HIFα-dependent pathway could be responsible for circadian disruption in OSA patients. Interestingly, it has been reported that the HIF1-α gene promotor exhibits an E-box sequence targeted by BMAL1:CLOCK [210]. Peek et al. revealed that disordered BMAL1 expression (which may contribute to circadian clock disruption [80]) results in an increased level of HIF-1α [211]. Moreover, BMAL1 gene silencing leads to reduced expression of HIF-1α. Therefore, it seems that the link between OSA and circadian clock disruption in an HIF-1α-dependent manner is bidirectional. However, further research is required to corroborate this relationship.

Additionally, microRNAs (miRNAs) can interact in the relationship between OSA and circadian clock disruption. These are short fragments of RNA, up to 30 nucleotides in size [212]. The role of these molecules is the regulation of the post-transcriptional silencing of targeted genes [213,214]. Interestingly, a single miRNA can influence the expression of many different genes. Therefore, these molecules are frequently involved in functional interaction pathways and the pathogenesis of many diseases [215,216]. Furthermore, it has been reported that the miRNA profile changes in hypoxic conditions. Some families of miRNAs respond to a low-oxygen environment, such as the miRNA-181 family [217]. Due to the IH conditions in OSA, hypoxia-susceptible miRNAs can play an important role in the pathogenesis and interactions of this disease. Hou et al. corroborated this hypothesis, revealing the upregulation of *miRNA-181* in patients suffering from OSA [218]. Interestingly, *miRNA-181* exhibits a connection with clock genes. It has been reported that *miRNA-181a-5p*, *-181b-5p*, *-181c-5p*, and *-181d-5p* target the *CLOCK*, *PER2*, and *PER3* genes [219]. Yang et al. showed that the abovementioned clock genes are downregulated at midnight in patients with severe OSA [220]. Based on these findings, impaired clock gene fluctuations caused by *miRNA-181* may lead to circadian clock disruptions.

Moreover, interactions between miRNA and sirtuins (SIRTs) can enhance this potential feedback loop. Sirtuins are class-III histone deacetylases, which are dependent on NAD+. Currently, seven SIRTs have been recognized. These enzymes have a significant impact on the regulation of human metabolism [190]. SIRTs regulate circadian rhythms by affecting both central and peripheral clocks [221]. Wang et al. confirmed the associations between SIRTs and clocks, showing that *SIRT1*-deficient mice presented alterations in *PER1*, *PER2*, *CRY1*, and *CRY2* gene expression [222]. One of the most probable molecular links triggering the above correlation is the deacetylation of BMAL1 by SIRT1 in the region of E-box sequencing. The consequence of this action is the downregulation of transcriptional activity of the CLOCK-BMAL1 complex. Furthermore, SIRT1 binds to this heterodimer and regulates the circadian rhythm [223]. Thus, changes in SIRT1 levels can lead to circadian clock disturbances, mediated by altered CLOCK-BMAL1 activation. Chen et al. revealed that the level of SIRT1 is decreased in OSA patients. Moreover, the application of nasal continuous positive airway pressure (CPAP) treatment, a gold standard in OSA treatment, results in the restoration of the blood concentration of this enzyme [224]. One of the miRNAs targeting *SIRT1* is the hypoxia-sensitive *miRNA-181* [219]. Thus, the downregulation of *SIRT1* caused by *miRNA-181* silencing could be a significant enhancement of the direct connection between miRNA and clock genes in the context of the development of circadian disruptions in OSA patients.

OSA is related to the upregulation of inflammatory responses [225]. Many studies have investigated the increased level of, e.g., tumor necrosis factor α (TNFα) [226], interleukin 6 (IL-6) [227], and interleukin 8 (IL-8) [228]. Among the most probable triggers of increased inflammatory responses in OSA patients is sympathetic activity following the activation of cellular immune pathways, as both IH and sleep fragmentation may drive a sympathetic system [229]. Additionally, TNFα and IL-6 exhibit daily rhythmicity, proving the relationship between cytokines and the core clock [230]. Patients suffering from OSA present TNFα rhythm alterations [231]. Furthermore, diurnal variations in TNFα, IL-6, and IL-8 in plasma in children with OSA have been observed [232]. Therefore, OSA is considered a chronic low-grade inflammatory disease. On the other hand, Li et al. revealed that an inflammation process leads to the disruption of circadian clock expression (BMAL1, CLOCK, PER1, PER2, NPAS2, and REV-ERB-α) in the brain and muscles of rats with neuroinflammation [233]. The correlation between systemic or neuroinflammation and OSA is still not fully understood. However, impaired cytokine levels in patients with OSA likely contribute to circadian clock disruptions.

Walton et al. suggested a mechanism responsible for a link between OSA and circadian clock disruption. IH increases the expression of HIF-1, which further drives metabolic changes, leading to decreased pH in the microenvironment. Acidification stimulates the spatial redistribution of lysosomes in cells. Acidity prevents the localization of the mechanistic target of rapamycin kinase (mTOR) to the lysosomal surface, leading to a decrease in circadian clock expression [234].

**Figure 4:**
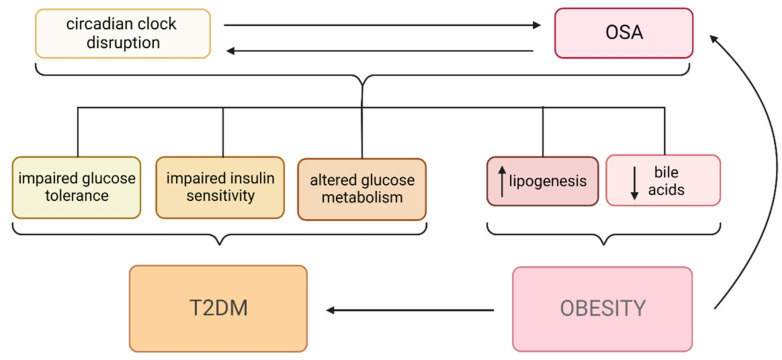
Summary of relationships between obstructive sleep apnea (OSA), circadian clock disruption, and its clinical effects. The relationship between circadian clock disruption and OSA is bidirectional. They both lead to variable metabolic changes—impaired glucose tolerance, impaired insulin sensitivity, and altered glucose metabolism—which result in the development of diabetes mellitus type 2 (T2DM). Increased lipogenesis and decreased bile acid levels promote the development of obesity. Obesity is also an independent risk factor for both OSA and T2DM. OSA—obstructive sleep apnea, T2DM—diabetes mellitus type 2.

The most important pathway in the opposite direction is related to metabolic disruption. As described above, circadian clock disruption leads to the development and aggravation of metabolic disorders [63]. Generally, the metabolic consequences of sleep and circadian rhythm disorders result in elevations in BMI and, frequently, the development of obesity [235,236]. Both are established risk factors for OSA development (see Figure 3 and Figure 4).

## 6. Limitation of the Study

Although we proposed to conduct a comprehensive review, this paper represents only a regular literature review, not a systemic review performed in accordance with the PRISMA guidelines. Therefore, it is characterized by lower transparency, accuracy, replicability, and a higher risk of biases. Moreover, the review was prepared by a young team with limited experience in sleep medicine, which may negatively affect its overall quality. Finally, there were limited high-quality data available pertaining to hypoxia-sensitive microRNA expression profiles in OSA patients. Therefore, the authors decided to focus on the best-described profiles—those of *miRNA181* and *miRNA199*.

## 7. Future Perspectives

Gaining better insights into the complex links between obstructive sleep apnea, circadian clock disruptions, and their metabolic consequences will lead to better diagnostic, prognostic, and treatment approaches in the future. The development of novel therapeutic options may rely on breaking this vicious circle, e.g., through minimizing the impact of hypoxia on the body and through the neutralization of unfavorable metabolic changes such as increased lipogenesis, altered glucose tolerance, and impaired insulin sensitivity. Moreover, the examination of microRNAs is becoming a cheaper and more reliable biomarker in this area of study. Therefore, it may become an important prognostic marker of OSA, as well as a possible treatment option for the affected pathways. Nevertheless, further studies are required to choose the best marker/target.

## 8. Conclusions

OSA is associated with an unfavorable metabolic profile and often leads to metabolic disorders such as diabetes mellitus, obesity, and non-alcoholic fatty liver disease. Many of the possible molecular pathways involved in this relationship have been described in the literature. In this review, we summarized the available data on the relationship between OSA and circadian clock disruption as a potential mechanism of metabolic disorders. Impaired circadian clock activity should be considered as an independent risk factor for metabolic diseases. The literature suggests that OSA-induced circadian clock disruption may be a potential signaling pathway that might be involved in the development and exacerbation of metabolic syndromes among OSA patients. Additionally, the bidirectional nature of the relationship requires consideration. Further studies on this topic are needed to help in the development of new therapeutic targets and the better selection of medications related to circadian clock targets.
